# Iatrogenic doramectin poisoning in newborn beef calves

**DOI:** 10.1007/s11259-026-11082-x

**Published:** 2026-01-30

**Authors:** Leandro da Silva Rocha, Wuglenya Daislla Martins da Silva, Larissa Sabino Pinho Moura, Ícaro Guilherme dos Santos, João Paulo da Silva Cavasani, Fernando Henrique Furlan, Edson Moleta Colodel

**Affiliations:** 1 Laboratório de Patologia Veterinária, Faculdade de Medicina Veterinária (FAVET), Universidade Federal de Mato Grosso (UFMT), Avenida Fernando Corrêa da Costa 2367, Boa Esperança, Cuiabá, Mato Grosso Brazil; 2 Clínica Veterinária, Instituto Federal de Educação, Ciência e Tecnologia de Rondônia (IFRO), Avenida Vereador Otaviano Pereira Neto 874, Setor 02, Jaru, Rondônia Brazil

**Keywords:** Macrocyclic lactones poisoning, Avermectin poisoning, Doramectin poisoning, Beef cattle

## Abstract

Doramectin is a safe antiparasitic drug widely used in animal production. However, two outbreaks of doramectin poisoning in newborn calves (*Bos indicus*) occurred after an accidental overdose of the drug was injected to prevent umbilical myiasis on a Brazilian farm. Affected calves exhibited clinical signs including apathy, sialorrhea, ataxia, and death. After a period of clinical manifestation lasting 24 to 48 h, a total of 91 calves died. Doramectin was found in brain samples from affected calves at a concentration exceeding the upper detection limit (25 µg/kg) of the quantification technique used. Diagnosis was based on history, clinical signs, the absence of significant gross and histopathological lesions, and the detection of doramectin in the brains of necropsied calves. These results highlight the importance of using doramectin with caution in newborn calves. Implementing practices such as weighing neonatal calves, especially leaner individuals, is critical to ensure accurate dosage calculations and to mitigate the risk of doramectin overdose.

## Introduction

Macrocyclic lactones, including avermectins (abamectin, ivermectin, eprinomectin, doramectin, and selamectin) and milbemycins (moxidectin, milbemycin, and nemadectin), are a group of structurally similar substances derived from natural compounds produced by soil-dwelling bacteria from the genus *Streptomyces*. These molecules belong to the macrocyclic lactones drug category, which exhibits insecticidal, acaricidal, and nematicidal activities (Merola and Eubig 2012; Salman et al. 2022), and have served as a fundamental milestone in parasite control since the late 1970 s (Shoop et al. 1995; Shoop and Soll 2002).

Although avermectins have a high margin of safety for their labeled indications in cattle (Merola and Eubig 2012; Shoop and Soll 2002; Rodrigues et al. 2018), cases of poisoning have been reported (Prichard et al. 2012). Abamectin is known to be the most toxic of the avermectins (Shoop and Soll 2002; Prichard et al. 2012), especially in calves with low body fat reserves or when administered at elevated doses (Seixas et al. 2006; Guizelini et al. 2020; Rocha et al. 2025). Rare cases of ivermectin toxicity (Udhavrao et al. 2017; Patel et al. 2018) and, most recently, doramectin toxicity (Machado et al. 2022; Souza et al. 2024) are also described in cattle.

The clinical condition involves neurological impairment, frequently with a fatal outcome (Lobetti et al. 2012; Nentwig et al. 2014; Cantón et al. 2022; Machado et al. 2022). Due to the absence of significant gross or histological findings (Lobetti and Caldwell 2012; Nentwig et al. 2014; Cantón et al. 2022; Machado et al. 2022; Souza et al. 2024), diagnosis is based on epidemiological data, clinical condition, and the detection of doramectin in tissues (Lobetti and Caldwell 2012; Cantón et al. 2022).

This study reports the epidemiological, clinical, and pathological findings of two outbreaks of doramectin poisoning in newborn beef calves.

### Case description and sample collection

Two disease outbreaks were recorded on a beef cattle farm located in the municipality of Ribeirão Cascalheira (12° 56′ 31″ S, 51° 49′ 26″ O), Mato Grosso state, in the Midwest region of Brazil, affecting newborn calves. The farm works as a beef cow-calf operation with 6,000 sows, estimated at 4,000 Nellore and 2,000 Angus or crossbreeds. Fixed-time artificial insemination is used to concentrate births towards the end of the dry season and the beginning of the rainy season in the Midwest region of Brazil (September-February).

The first outbreak occurred in February 2023, when the use of a long-acting endectocide based on 3.5% doramectin (Treo ACE^®^ - Zoetis) was used to prevent umbilical parasitic infestations (myiasis) and ticks. The medication was recommended at a dose of 700 µg/kg of body weight (BW) (1 ml per 50 kg BW) administered subcutaneously, for calves born during the season and over 30 days old, according to the manufacturer’s recommendations. However, during farm health management on the farm, it was decided to use 2 ml per calf, including newborns. During this management, no other medication was administered, and the calves received no additional treatment. The group consisted of 3000 calves, constituted by approximately 1950 Nellore (*Bos indicus*) and 1050 Angus (*Bos taurus*) or crossbreeds. Their ages ranged from newborn to 4 months, and their estimated weights ranged from 22 kg to 150 kg.

Of the 272 newborn calves treated, 96 Nellore calves became ill, of which 71 died and 25 recovered. Clinical signs included apathy, sialorrhea, motor incoordination, and death. These signs appeared between 24 and 48 h after administration. The clinical course ranged from a few hours to a week. After this occurrence, drug poisoning was suspected, and the medication to control umbilical myiasis was changed to 1% ivermectin (Ivomec^®^ - Merial) and was used in another batch of newborn calves. However, its use was discontinued due to insufficient clinical efficacy.

In March 2023 (the second outbreak), the day after the birth of 45 calves, 3.5% doramectin (Treo^®^ ACE - Zoetis) was again used, with 1 ml applied per calf, regardless of birth weight. Of these, 25 became ill, 20 died, and five recovered. The clinical signs were similar to those observed in the previous occurrence.

During clinical evaluation, the calf’s body condition score (BCS) was estimated on a scale of 1 (thin) to 9 (fat), as proposed by Kunkle et al. (1994). In both outbreaks, only Nellore calves, born weighing between 22 and 25 kg (BCS = 3), were affected by doramectin intoxication at doses ranging from 1,400 to 3,181 µg/kg BW (2 to 4,5 times the manufacturer’s recommended dose). There were no reports of clinical changes in Angus and Angus-crossbred calves (BCS = 5).

Two Nellore calves, one from each outbreak (one that died spontaneously and the other that was euthanized *in extremis*), were necropsied; tissue samples were collected, fixed in 10% formalin, and routinely processed for microscopic examination at the Veterinary Pathology Laboratory of the Federal University of Mato Grosso.

Samples from the central nervous system of each calf were collected, frozen at − 20 °C, and sent in dry ice for the determination of avermectin concentrations in the tissues using liquid chromatography with tandem mass spectrometry (LC-MS/MS), with validation and a limit of quantification of 2.5–25 µg/kg, at the Microbioticos^®^ Laboratory in Campinas, São Paulo, Brazil. The concentration of doramectin was analyzed in the samples, and abamectin was also included due to the similarity of clinical signs observed in cattle intoxicated by abamectin in Mato Grosso state (Rocha et al. 2025).

No significant macroscopic or microscopic alterations were found in any of the evaluated tissues. Doramectin was detected in the brain sample from the necropsied calves at a concentration exceeding the upper detection limit (25 µg/kg) of the quantification technique used, while abamectin was absent.

## Discussion

In these cases, the diagnosis was based on the injectable dose of doramectin administered, the calves’ body weight, clinical signs, the absence of significant morphological changes, and the detection of the active ingredient in the central nervous systems (CNS) of the calves that developed clinical signs.

Apathy, sialorrhea, and incoordination progressing to a fatal outcome with the absence of postmortem lesions, as noted in this study, are similar to others’ descriptions of doramectin poisoning in cattle (Machado et al. 2022; Souza et al. 2024), sheep (Cantón et al. 2022), lions (Lobetti and Caldwell 2012), and dog (Yas-Natan et al. 2003), as well as, in the poisoning by other avermectins, especially abamectin (Seaman et al. 1987; Button et al. 1988; Seixas et al. 2006; Guizelini et al. 2020; Borges et al. 2021; Rocha et al. 2025).

The primary justification for the use of avermectins on this farm was the prevention of umbilical myiasis in newborn calves. *Cochliomyia hominivorax*, the New World screwworm, is recognized as the principal etiological agent of primary myiasis affecting livestock, companion animals, and humans in Brazil (Costa-Júnior et al. 2019). However, the preventive efficacy of injectable drugs used to control umbilical myiasis in newborn beef calves remains limited (Aquino et al. 2022). Reports indicate resistance of *C. hominivorax* to 1% doramectin, as well as reduced effectiveness of 3.15% ivermectin in preventing infestations caused by this species, reinforcing the need for more effective control strategies (Muchiut et al. 2024).

Avermectins usually do not cause toxic effects in adult cattle because they are molecules with high molecular weight (850–900 Da) and due to the action of membrane efflux pumps in the endothelium of vessels from the blood-brain barrier (BBB). The tight gap between the cells forming the BBB allows passive diffusion of lipid-soluble drugs at molecular weights lower than 400–600 Da (Kadry et al. 2020; Wu et al. 2023). However, due to their high lipophilicity, some high-molecular-weight drugs, such as avermectins, may still cross the BBB (Prichard et al. 2012; Lespine 2013; Batiha et al. 2020; Wu et al. 2023). When this occurs, the drugs are transported out of the endothelial cells back into the blood circulation by membrane efflux pumps belonging to the ABC (ATP-binding cassette) transporter family, mainly P-glycoprotein (P-gp) (Lespine 2013; Mallard et al. 2018).

The ABC transporter family consists of transmembrane proteins that efflux a wide variety of amphiphilic compounds with diverse chemical structures out of cells and organisms. Thus, they provide an efficient protective barrier and limit the entry of many drugs, restricting their toxicity (Prichard et al. 2012; Lespine 2013; Batiha et al. 2020). P-gp is considered the most important ABC transporter in the BBB regarding avermectin access to the brain (Geyer et al. 2009; Merola and Eubig 2012; Mealey et al. 2023). It was demonstrated that animals with P-gp deficiency, due to low gene expression or gene deletion, exhibit increased sensitivity to avermectin neurotoxicity (Geyer et al. 2007; Kiki-Mvouaka et al. 2010; Merola and Eubig 2012).

Although avermectins are generally considered safe for adult ruminants (Prichard et al. 2012; Lespine 2013; Batiha et al. 2020; Rodrigues et al. 2018), there are some outbreaks reporting young animals (especially up to four months old) exhibited severe intoxication characterized by neurological signs (Button et al. 1988; Seixas et al. 2006; Guizelini et al. 2020; Machado et al. 2022; Souza et al. 2024) as described in this study, with detection of avermectins in the central nervous system (Borges et al. 2021; Cantón et al. 2022; Rocha et al. 2025). However, older animals may also be affected (Seaman et al. 1987; Cantón et al. 2022; Machado et al. 2022; Rocha et al. 2025).

Some authors believe it occurs due to immaturity or increased BBB permeability in young animals (Button et al. 1988; Nentwig et al. 2014; Guizelini et al. 2020). However, a critical examination of relevant scientific literature shows that many concepts of the BBB are incorrect. These include the belief in its immaturity that is unfortunately often equated with absence or at least leakiness in the embryo, fetus, newborn, and calves. The BBB is fully morphologically formed at birth, and it is incorrect to claim that there is a generalized increase in permeability; this occurs only for particular substances, such as avermectins (Saunders et al. 2014; Mallard et al. 2018).

The therapeutic safety of avermectins in adult animals is related to the level of expression and activity of the gene for the multidrug-resistant protein 1 (MDR1 or ABCB1) that encodes for P-gp in the BBB (Geyer and Janko 2012; Lespine 2013; Mallard et al. 2018). Toxic effects and detection of avermectins in the CNS are observed when blood concentrations are high enough to saturate clearance by the P-gp efflux pump (Van Amstel et al. 2009; Borges et al. 2021; Cantón et al. 2022). These effects are more severe in newborn animals because, although the BBB is morphologically completely formed, and the expression of the ABC transporter genes and proteins is effective in other organs, specifically, in the vessels of the CNS, the MDR1 and P-gp expression is very low at birth and increases significantly over the next two weeks (Gazzin et al. 2008; Ek et al. 2010; Mallard et al. 2018; Merola and Eubig 2012; Verscheijden et al. 2020).

Avermectins are usually not detected in the CNS. It occurs under specific circumstances, such as overdosing or use in animals with poor body condition (scarce adipose tissue) (Seaman et al. 1987; Seixas et al. 2006; Guizelini et al. 2020; Cantón et al. 2022; Machado et al. 2022; Souza et al. 2024; Rocha et al. 2025). In this study, newborn Nellore calves weighing between 22 and 25 kg became ill or died after receiving 1 to 2 ml of 3.5% doramectin, totaling doses ranging from 1,400 to 3,181 µg/kg BW, 2 to 4.5 times the manufacturer’s recommended dose (700 µg/kg BW). These values are higher than the margin of safety to adult cattle (1,000 µg/kg BW); however, they are lower than the values of drug tolerance for adult cattle (5,000 µg/kg BW) (Vercruysse and Rew 2002). Curiously, the manufacturer’s recommended dose is higher than the dose tested in a neonatal safety study performed with calves (600 µg/kg BW). This is probably due to the high drug tolerance value observed in cattle.

Additionally, based on the body condition of the animals in this study, the calves were lean and below the average weight (29.8 kg) for the Nellore breed (Nobre et al. 2003). Long-acting avermectin formulations (3.5%) replace traditional formulations (1%). These drugs were designed for cattle, and the vehicle plays a fundamental role in forming a depot at the subcutaneous site and thus releasing the drug slowly to achieve greater persistence (Lifschitz et al. 2007; Cantón et al. 2022). We hypothesize that in the calves in this study, this deposit was not formed, such that the animals had poor body condition and scarce adipose tissue, a large quantity of the drug was rapidly bioavailable in the plasma, achieving high concentrations that crossed the BBB, saturating the activity of P-gp and worsening the overdose. The impact of fat body status on the pharmacokinetics of macrocyclic lactones has been described in different species (Echeverría et al. 2002; Lespine 2013; Bousquet-M'elou et al. 2021), and its interaction with overdosing has been proposed (Merola and Eubig 2012; Machado et al. 2022; Cantón et al. 2022).

Calf weight can be influenced by factors such as breed, time of year of birth (Forster et al. 2010), sex of the calf, and age of the cow at calving (Alencar et al. 1999). In this case, the dose of doramectin for newborns was standardized (1 or 2 ml per calf), and the poisoning occurred exclusively in Nellore calves. Calves from Nellore cows tend to have a lower average birth weight than those from European and mixed breeds. Additionally, Nellore cows had lower milk production (than European breed cows), which may be associated with the slower deposition of fat reserves (Alencar et al. 1998). These factors may have increased the breed’s susceptibility to poisoning, as observed in this study.

In conclusion, the administration of higher doses of doramectin to calves with poor body condition and scarce adipose tissue can cause severe intoxication characterized by neurological signs with no significant morphological changes, and the detection of doramectin in the CNS of the calves that developed clinical signs. Failure to accurately determine animal weight prior to dosing, especially in cases of poor body condition, represents the leading risk factor for doramectin overdose.

## Data Availability

No datasets were generated or analysed during the current study.
